# A Case of Reconstruction Using a Nerve Conduit After Total Resection of Digital Nerve Lipomatosis

**DOI:** 10.7759/cureus.93370

**Published:** 2025-09-27

**Authors:** Mika Akahane, Akari Mori, Kensho Suzuki, Kaoru Tada, Satoru Demura

**Affiliations:** 1 Orthopaedic Surgery, Graduate School of Medical Science Kanazawa University, Kanazawa, JPN

**Keywords:** digital nerve, fibrolipomatous hamartoma, lipomatosis of nerve, nerve conduit, nerve reconstruction, total resection

## Abstract

Lipomatosis of the nerve in the digital nerve is extremely rare. We report a case of lipomatosis of the nerve in the digital nerve on the ulnar side of the ring finger in a 20-year-old female. The patient presented with neurological symptoms, including pain and numbness. Magnetic resonance imaging revealed a fatty lesion on the ulnar side of the left ring finger, and biopsy revealed adipose tissue and fibrous stroma, but no definitive diagnosis was made. Surgical treatment consisted of total excision of the fatty tumor along with the affected nerve, followed by nerve reconstruction using a nerve conduit, as decompression or partial resection was not feasible. Histopathological examination confirmed the diagnosis of fibrolipomatous hamartoma. No progression or new lesions were detected at three‑year follow‑up. Sensory function on the ulnar side of the ring finger improved, and range of motion remained normal. When encountering a fatty soft tissue tumor around a nerve, the possibility of lipomatosis of the nerve should be considered, and surgical planning should take into account the potential need for nerve resection and reconstruction.

## Introduction

Lipomatosis of nerve (LN), also referred to as fibrolipomatous hamartoma, is a rare, benign hamartomatous lesion characterized by the proliferation of fibrofatty tissue within the epineurium of peripheral nerves. Unlike true neoplasms, LN is a congenital malformation rather than a neoplastic growth. The condition was first described by Mason [1] in 1953, and more than 100 cases have been reported to date. More than 80% of these cases involve the median nerve at the carpal tunnel while cases involving the digital nerve are extremely rare [2]. LN is associated with macrodactyly in about 30% of cases. Macrodactyly is a congenital enlargement of the fingers characterized by the overgrowth of all mesenchymal elements, including fibrofatty tissue, bone, and other connective tissues. LN is often considered part of this spectrum, as its histological features, namely, fibrofatty proliferation between nerve bundles, are consistent with those observed in macrodactyly [2,3]. It is regarded as a congenital lesion because it frequently presents early in life [4].

Clinically, LN may remain asymptomatic in early stages but often leads to progressive symptoms of entrapment neuropathy, such as numbness, pain, and weakness [5]. Diagnosis can be challenging, especially when typical imaging features are absent, as is often the case with digital nerve involvement [2]. Furthermore, surgical decision-making is complex due to the delicate balance between symptom relief and potential loss of nerve function. Treatment strategies range from conservative management to decompression, limited debulking, or even complete nerve resection with reconstruction, each with distinct risks and benefits [3,4]. Differential diagnosis of LN occurring in the fingers includes Schwannoma, neurofibroma, ganglion cysts, hemangiom, fibromatosis and lipomas. These lesions may present with similar clinical features such as a palpable mass or pain, and distinguishing them preoperatively can be challenging, especially when typical radiological findings are absent [3,6,7,8].

In this study, we report a case of LN involving the digital nerve of the ring finger, in which total resection and nerve reconstruction with a nerve conduit were performed. This case highlights the diagnostic difficulties and intraoperative challenges encountered when the tumor is inseparable from the nerve, and underscores the importance of thorough preoperative planning in managing rare intraneural lesions.

## Case presentation

**Figure 1:**
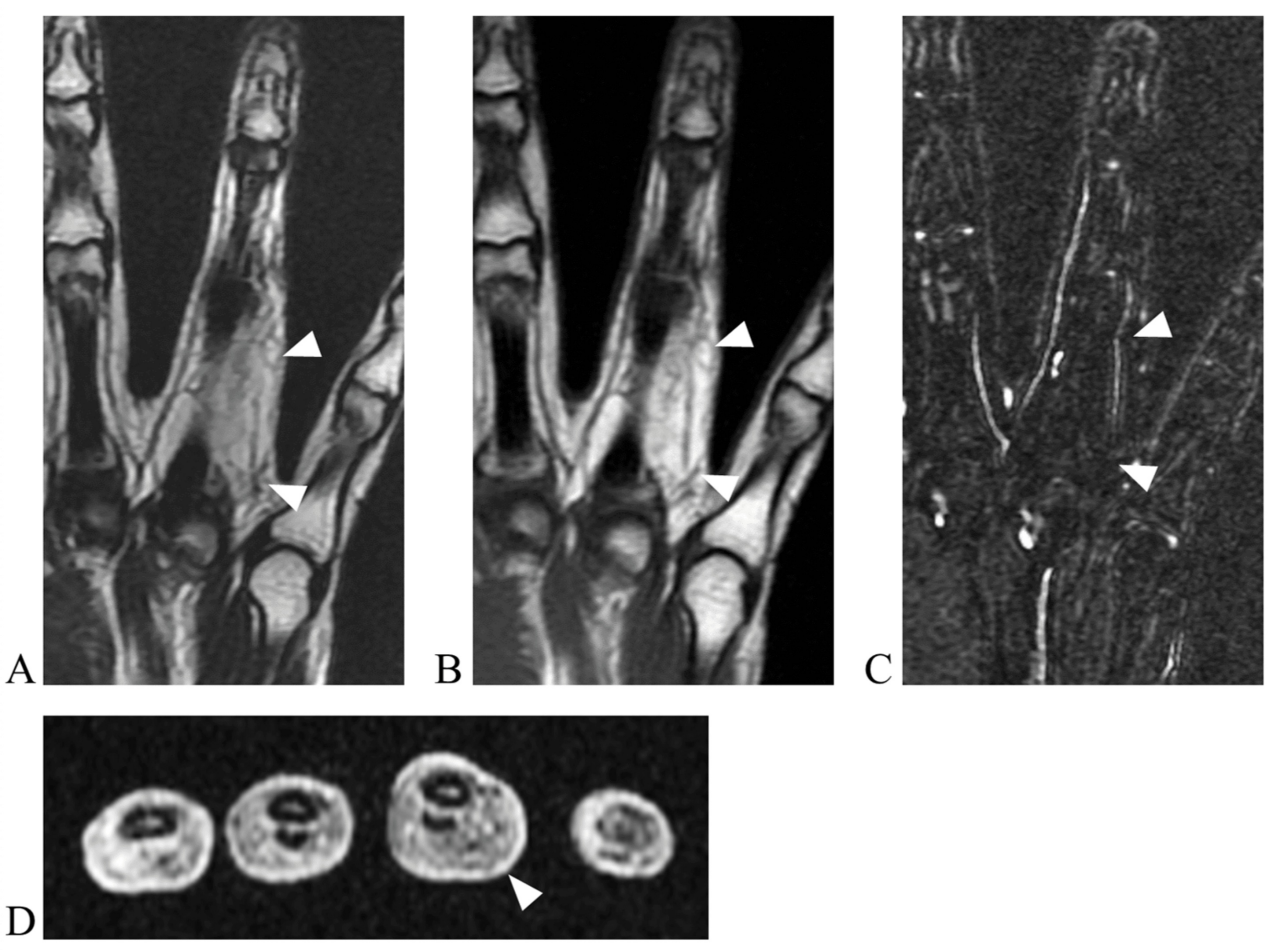
Magnetic resonance imaging demonstrating a fatty lesion on the ulnar side of the left ring finger (white arrow). (A) Sagittal section, T1-weighted images; (B) sagittal section, T2-weighted images; (C) sagittal section, fat-suppressed image; (D) axial section, T1-weighted images.

**Figure 2:**
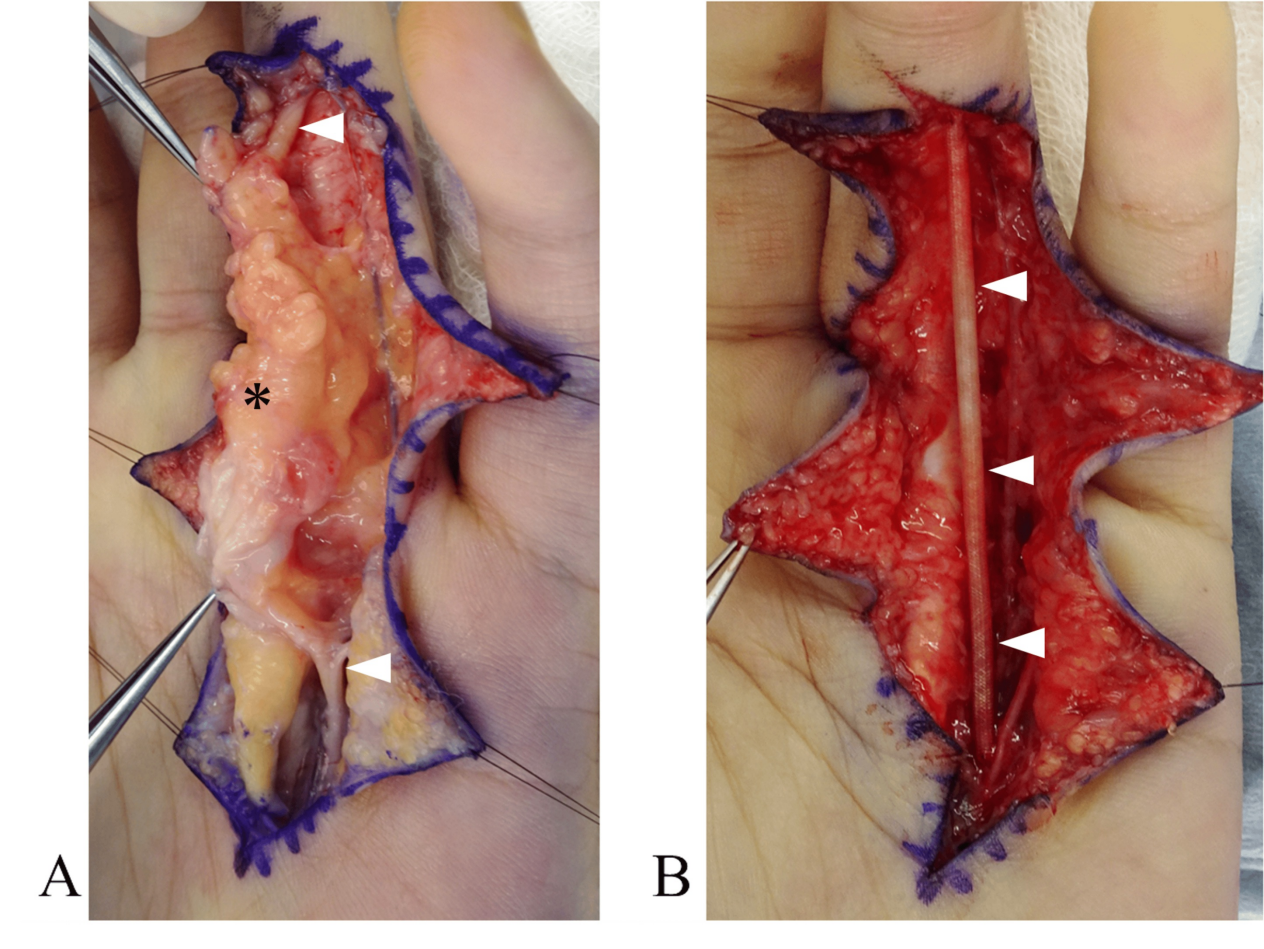
Intraoperative photographs. (A) A fat-like tumor (black asterisk), adherent to the surrounding tissues, was found to originate from the digital nerve (white arrowhead). (B) A nerve conduit (white arrowhead) was implanted at the site of the nerve defect.

**Figure 3:**
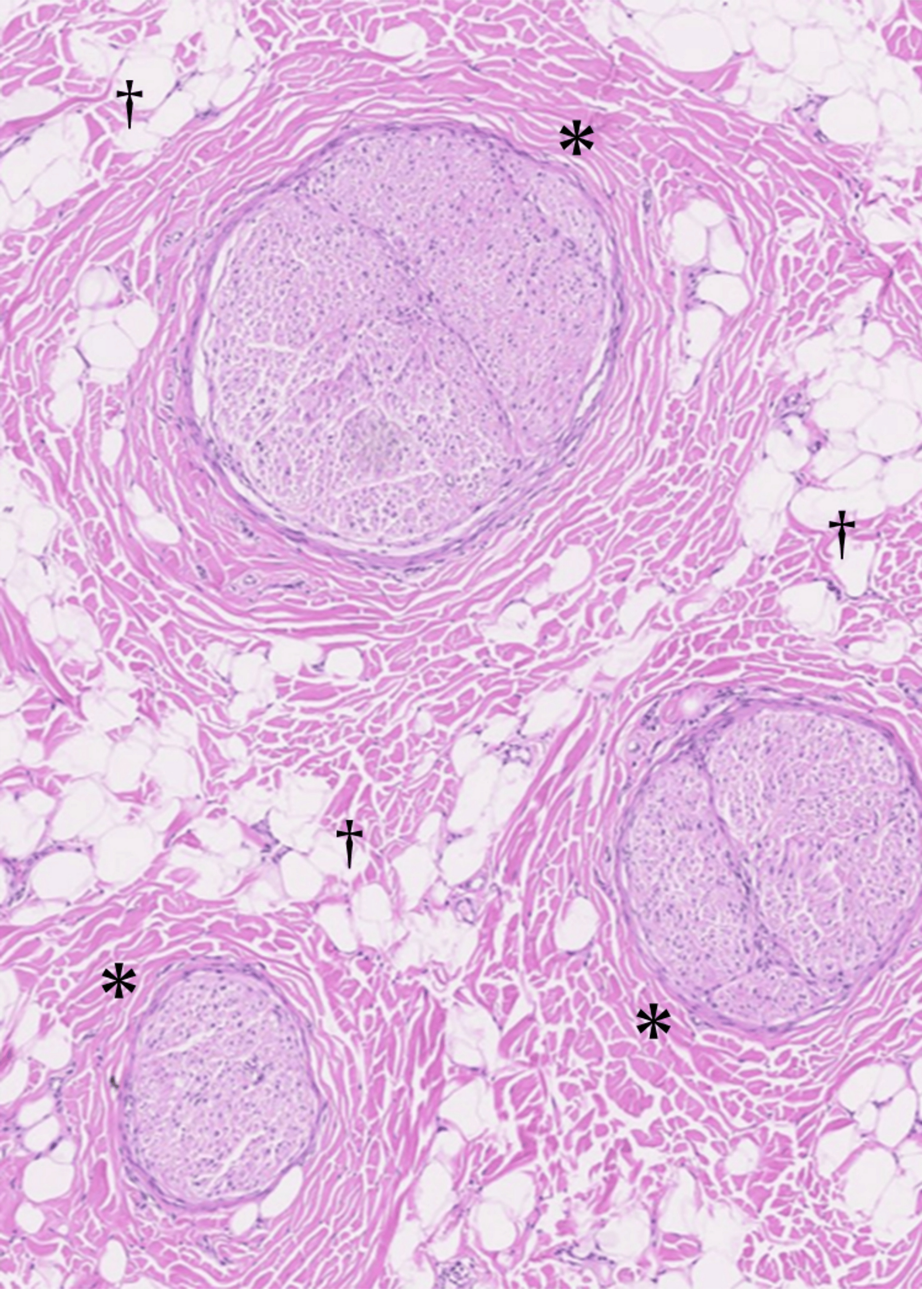
Histopathological finding. Histopathological examination showing features consistent with fibrolipomatous hamartoma. Nerve bundles were surrounded by fibrous tissue (black asterisk) and hyperplasia of fibroadipose tissue (black dagger) within the epineurium.

The patient was a 20-year-old female, a nursing student, and right-hand-dominant, who was aware of a mass on her left ring finger for several years. The tumor grew slowly. She came to our department when she became aware of pain and numbness in her finger. At the initial visit to our department, a soft mass was palpated on the left ring finger, extending from the palm to the proximal interphalangeal joint. Numbness occurred on the ulnar side of the finger when the tumor was compressed. Magnetic resonance imaging (MRI) revealed a fatty lesion on the ulnar side of the left ring finger with high signal on T1-weighted images, high signal on T2-weighted images, and fat suppression on fat-suppressed images (Figure 1). An incisional biopsy revealed adipose tissue and fibrous stroma, but no definitive diagnosis was made. Since no malignant findings were observed, lipoma was the primary preoperative diagnosis, and resection was planned. Before surgery, the patient was informed that, if the lesion was found to involve the digital nerve and was inseparable from it, partial or total nerve resection followed by reconstruction might be required. Written informed consent for possible nerve resection and reconstruction was obtained. Surgical examination revealed a fat-like tumor that was adherent to surrounding tissues and had indistinct borders. Further dissection revealed that the tumor originated from the digital nerve on the ulnar side of the ring finger (Figure 2A). The tumor could not be separated from the nerve even under microscopy due to intraneural infiltration. Fibrofatty tissue was interspersed between and around the fascicles, making nerve preservation unfeasible. Given the diffuse and inseparable nature of the lesion, decompression or debulking would have risked damaging the nerve. Therefore, total resection of the tumor, including the nerve, was deemed necessary. A nerve conduit (1.5 mm × 55 mm, Nerbridge, Toyobo Co., Ltd., Japan) was implanted at the site of the nerve defect (Figure 2B). Histopathological examination revealed nerve bundles surrounded by fibrous tissue and hyperplasia of fibroadipose tissue in the epineurium (Figure 3), and the patient was diagnosed with fibrolipomatous hamartoma. No progression or new lesions were detected at three‑year follow‑up. Sensory on the ulnar side of the ring finger improved to 3.22 (blue) in the Semmes-Weinstein test. The static two-point discrimination was 4 mm (3 mm on the radial side of the ring finger). A hypertrophic scar was formed at the wound site, but the range of motion was normal, and the Disability of the Arm, Shoulder, and Hand (DASH) score was zero.

## Discussion

LN is a benign hamartomatous lesion characterized by fibrofatty proliferation within the epineurium. It is known under different names, such as fibrolipomatous hamartoma, lipofibromatous hamartoma, fatty infiltration of the nerve, neural lipofibroma, intraneural lipoma, and lipofibroma [2,3,9,10,11]. LN typically affects the median nerve at the carpal tunnel, where it exhibits characteristic MRI features such as a “coaxial cable-like” appearance on axial views and a “spaghetti-like” fascicular appearance on coronal views [2]. Therefore, LN can often be diagnosed via MRI, and biopsy is not always necessary for definitive diagnosis [3,4]. However, such findings are characteristic of LN in the median nerve, and typical MRI features are often absent in cases involving the digital nerves, making diagnosis by MRI alone more difficult [2]. In our case as well, the MRI did not show the typical appearance of LN and was difficult to diagnose. Therefore, biopsy and resection were planned.

Table 1 summarizes representative cases of lipomatosis of nerve involving the digital nerves from the literature, including the present case. Clinically, LN is asymptomatic in the early stages but slowly progresses over several years with neurological symptoms such as pain and numbness, hypersensitivity, and muscle weakness [5]. If no neurological symptoms are present, conservative treatment is recommended [3,4]. If neurological symptoms are present, decompression, partial resection of the tumor [3,11], or total resection of the tumor, including the nerve [3,7,12] has reportedly been conducted. However, because tumor resection results in loss of nerve function, it should be performed only when conservative therapy or decompression does not improve symptoms and the surgical benefits outweigh the risks [3,4]. Moreover, Intraneural lipomas and fibrolipomatous hamartoma (lipofibromatous hamartomas or lipofibroma) are included in LN, but differ significantly in pathology and management. Intraneural lipomas are well-demarcated and noninvasive, often allowing for complete excision without nerve sacrifice. In contrast, fibrolipomatous hamartoma are infiltrative, enveloping nerve fascicles, and frequently require nerve resection and reconstruction [13].　

In the present case, the patient presented with neurological symptoms, but decompression was not possible, and partial resection could damage the nerve. Therefore, total resection of the tumor, including the nerve, was performed, and reconstruction with a nerve conduit was performed at the site of the nerve defect. However, in general, nerve conduits are indicated for thin sensory nerve defects of 30 mm or less [14,15], and we consider that it was inappropriate to adapt nerve conduits to this case. Sensory in the finger pulp is also controlled by the contralateral digital nerve, superficial branch of the radial nerve, and dorsal branch of the ulnar nerve [16], and even in the case of unilateral digital nerve injury, there may still be sensory receptors in the finger pulp that have not been denervated. The recovery of sensory in the present case is unlikely to be caused by nerve regeneration with the nerve conduit, and sensory innervation from the contralateral digital nerve and postoperative sensory re-innervation from the surrounding nerve branches may have improved the Semmes-Weinstein test score and the static two-point discrimination. Normally, autologous nerves or processed nerve allografts should be the first choice when tumor resection or other procedures result in large nerve defects. In the case of a fatty soft tissue tumor around the nerve, LN should have been included for differential diagnosis, and the pre-operative planning, including autologous nerve grafts or processed nerve allografts, should have been performed before surgery. While differential diagnoses of tumors around digital nerves, such as schwannoma, neurofibroma, and lipoma, deserve further clinical attention due to their overlapping presentations and varying management strategies. LN, although rare, can present without classic imaging signs, especially in the digital nerves [2]. This case reinforces the importance of not only considering LN in the differential diagnosis of fatty soft tissue tumors of the digits, but also of preparing for complex intraoperative decisions such as nerve grafting or reconstruction.

**Table 1:** Representative cases. Comparison of management strategies and outcomes in reported cases of lipomatosis of nerve involving digital nerves.

Author (Year)	Location (Nerve)	Symptoms	Preoperative/postoperative diagnosis	Surgery type	Reconstruction	Outcome
Jung et al. (2005) [8]	Digital nerve of the bilateral index fingers	Asymptomatic	No examination/lipofibromatous hamartoma	Partial resection (The nerve was preserved.)	None	No recurrence, no sensory dysfunction
Nanno et al. (2011) [2]	Digital nerve of the left thumb	Asymptomatic	Lipoma/fibrolipomatous Hamartoma	Partial resection (The nerve was preserved)	None	No recurrence, no neurological deficit
DeSano J 2nd et al. (2021) [13]	Digital nerve of the right ring finger	Pain, paresthesias	Intraneural lipoma/intraneural lipoma	Dissection (The nerve was preserved)	None	Paresthesias resolved
Su and Bhandari (2022) [6]	Digital nerve of the right ring finger	Cold intolerance	Lipoma/intraneural Lipoma	Microdissection (The nerve was preserved)	None	No recurrence, Intact sensation, diminishing cold intolerance
Present case	Digital nerve of the left ring finger	Pain, numbness	Lipoma/fibrolipomatous hamartoma	Total resection	Nerve conduit	No recurrence, Sensory improved

## Conclusions

Given the potential diagnosis of LN, surgeons should carefully evaluate fatty soft tissue tumors involving peripheral nerves, with preoperative planning that considers the possible necessity of nerve resection and reconstruction. In particular, since LN in the digital nerve can be difficult to diagnose by MRI alone, it is essential to consider histological confirmation before definitive treatment. Moreover, when nerve resection is unavoidable, appropriate preparation, including options for nerve grafting or alternative reconstruction methods, is crucial to optimize functional outcomes.
